# Root cone angle is enlarged in *docs1* LRR-RLK mutants in rice

**DOI:** 10.1186/s12284-017-0190-1

**Published:** 2017-12-15

**Authors:** M. Bettembourg, M. Dal-Soglio, C. Bureau, A. Vernet, A. Dardoux, M. Portefaix, M. Bes, D. Meynard, D. Mieulet, B. Cayrol, C. Perin, B. Courtois, J. F. Ma, A. Dievart

**Affiliations:** 1 CIRAD, UMR AGAP, F34398 Montpellier, France; 2 AGAP, Univ Montpellier, CIRAD, INRA, Montpellier SupAgro, Montpellier, France; 3 Institute of Plant Science and Resources, Okayama University, Chuo 2-20-1, Kurashiki, 710-0046 Japan; 4 Present address: Shanghai Jiao Tong University (SJTU), School of Life Sciences and Biotechnology, Shanghai, 200240 China

**Keywords:** Rice, Root cap, Angle, Gravitropism, docs1, Lrr, Rlk, GSA, RCA, RGA

## Abstract

**Background:**

The *DEFECTIVE IN OUTER CELL LAYER SPECIFICATION 1* (*DOCS1)* gene belongs to the Leucine-Rich Repeat Receptor-Like Kinase (LRR-RLK) subfamily. It has been discovered few years ago in *Oryza sativa* (rice) in a screen to isolate mutants with defects in sensitivity to aluminum. The c68 (*docs1–1*) mutant possessed a nonsense mutation in the C-terminal part of the DOCS1 kinase domain.

**Findings:**

We have generated a new loss-of-function mutation in the *DOCS1* gene (*docs1–2*) using the CRISPR-Cas9 technology. This new loss-of-function mutant and *docs1–1* present similar phenotypes suggesting the original *docs1–1* was a null allele. Besides the aluminum sensitivity phenotype, both *docs1* mutants shared also several root phenotypes described previously: less root hairs and mixed identities of the outer cell layers. Moreover, our new results suggest that DOCS1 could also play a role in root cap development. We hypothesized these *docs1* root phenotypes may affect gravity responses. As expected, in seedlings, the early gravitropic response was delayed. Furthermore, at adult stage, the root gravitropic set angle of *docs1* mutants was also affected since *docs1* mutant plants displayed larger root cone angles.

**Conclusions:**

All these observations add new insights into the *DOCS1* gene function in gravitropic responses at several stages of plant development.

**Electronic supplementary material:**

The online version of this article (10.1186/s12284-017-0190-1) contains supplementary material, which is available to authorized users.

## Findings

Plant root systems show usually complex architectures, with most roots growing vertically (i.e. negative orthogravitropism) but some also growing more radially (i.e. plagiogravitropism) into the ground. The gravitropic equilibrium position at which organs will grow has been called the gravitropic set-point angle (GSA) (Digby and Firn 1995; Firn and Digby 1997). In fibrous root systems, like that of rice, the GSA of the shallowest crown roots is an important root parameter because it depicts well the global shape of the root system. To report this root system parameter, the root cone angle (RCA) or the root growth angle (RGA) are usually measured. The RCA is the angle between the two most external right and left roots in a two-dimensional system. The RGA is the angle between the soil surface and the shallowest crown root (Kitomi et al. 2015). These two measures are then supplementary (2 RGA + RCA = 180°). It is assumed that the degree of RCA openness or narrowness could have a strong effect on the ability of plants to explore their external surrounding to optimize their resource uptake (Lynch 2013; Uga et al. 2015). For example, an open RCA could impact the uptake efficiency of nutrient such as phosphate, which accumulates preferentially in the topsoil.

To date, two genes affecting RGA have been cloned in rice: *DRO1* (*DEEPER ROOTING 1*) and *SOR1* (*SOIL-SURFACE ROOTING 1*) (Hanzawa et al. 2013; Uga et al. 2011; Uga et al. 2013). Both genes could affect auxin signal transduction during the gravitropic response. This suggests that polar auxin transport during the gravitropic response is an important determinant of the RGA in rice plants, as it is in Arabidopsis. Indeed, it has been reported recently in Arabidopsis that regulation of polar auxin redistribution determined GSA in lateral roots (Rosquete et al. 2013). The high or low auxin level/signaling resulted in an axial or radial root system, respectively. Not surprisingly, the cellular and molecular mechanisms leading to auxin redistribution in cells and tissues for GSA establishment rely in part on the same actors than the ones involved in gravity response. First, gravity sensing depends on statoliths relocalization in columella cells. Then, relocalization of auxin efflux-carrier proteins on cell membranes will generate asymmetric, directional, specific and localized auxin flows across cells and tissues (Adamowski and Friml 2015). These polar auxin gradients will lead to differential root side cell expansion to activate root curvature (Armengot et al. 2016). The fine regulation of these spatio-temporal auxin fluxes will then be of great importance for root system architecture establishment and environmental adaptive responses.

The *DEFECTIVE IN OUTER CELL LAYER SPECIFICATION 1* (*DOCS1*) gene belongs to the large family of leucine-rich repeat receptor-like kinases (LRR-RLKs) involved in many developmental and environmental responses (Wu et al. 2016). In rice, a mutant of this gene, named c68, carries a nonsense mutation in the kinase domain (Huang et al. 2012). The c68 mutant has been discovered in a screen for aluminum sensitivity (Huang et al. 2009). In aluminum-rich media, root elongation of c68 plants was inhibited compared to that of the Koshihikari wild type (WT) plants. The c68 mutant plants also showed several root phenotypes: a reduced number and size of root hairs, and layers of external tissues with exodermis/epidermis mixed identity (Huang et al. 2009).

In this study, we have created a null allele of *DOCS1* and have shown that mutant plants exhibited impaired gravity responses at different developmental stages. At 3 days, a delay of response to gravity was observed during the first hour after gravistimulation. At 30 days, we observed that the RCA of mutant plants was more open than that of WT plants. Our new data suggest that the DOCS1 receptor could be involved in the auxin response at several stages of development. Root cap morphology and cell identity defects observed in several root external tissues may affect auxin efflux and influx transport in root tip following root gravistimulation, or at later stages during GSA establishment.

## Results

### *docs1–1* is a null allele

**Fig. 1:**
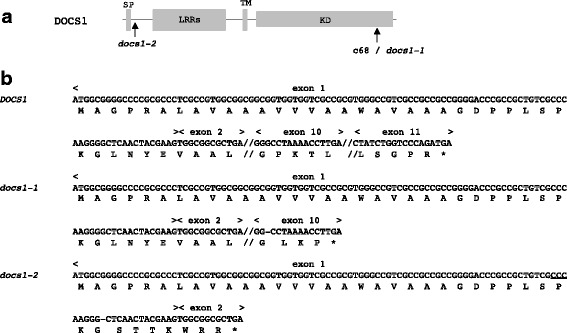
Allelic variants of *DOCS1*. **a** Schematic representation of the DOCS1 receptor with positions of the CRISPR targeted (*docs1–2*) and *docs1–1* mutations. **b** The nucleotide and protein sequences of the *DOCS1* gene and the two allelic variants, *docs1–1* and *docs1–2*, are represented. The new allelic variant *docs1–2* presents a deletion of one nucleotide 95 pb after the START codon, at the end of the 1st exon. The PAM sequence located 3 nucleotides upstream of the double strand break site is underlined. The deletion of one nucleotide in *docs1–1* is located in the 10th exon. Bars represent nucleotide deletions

**Fig. 2:**
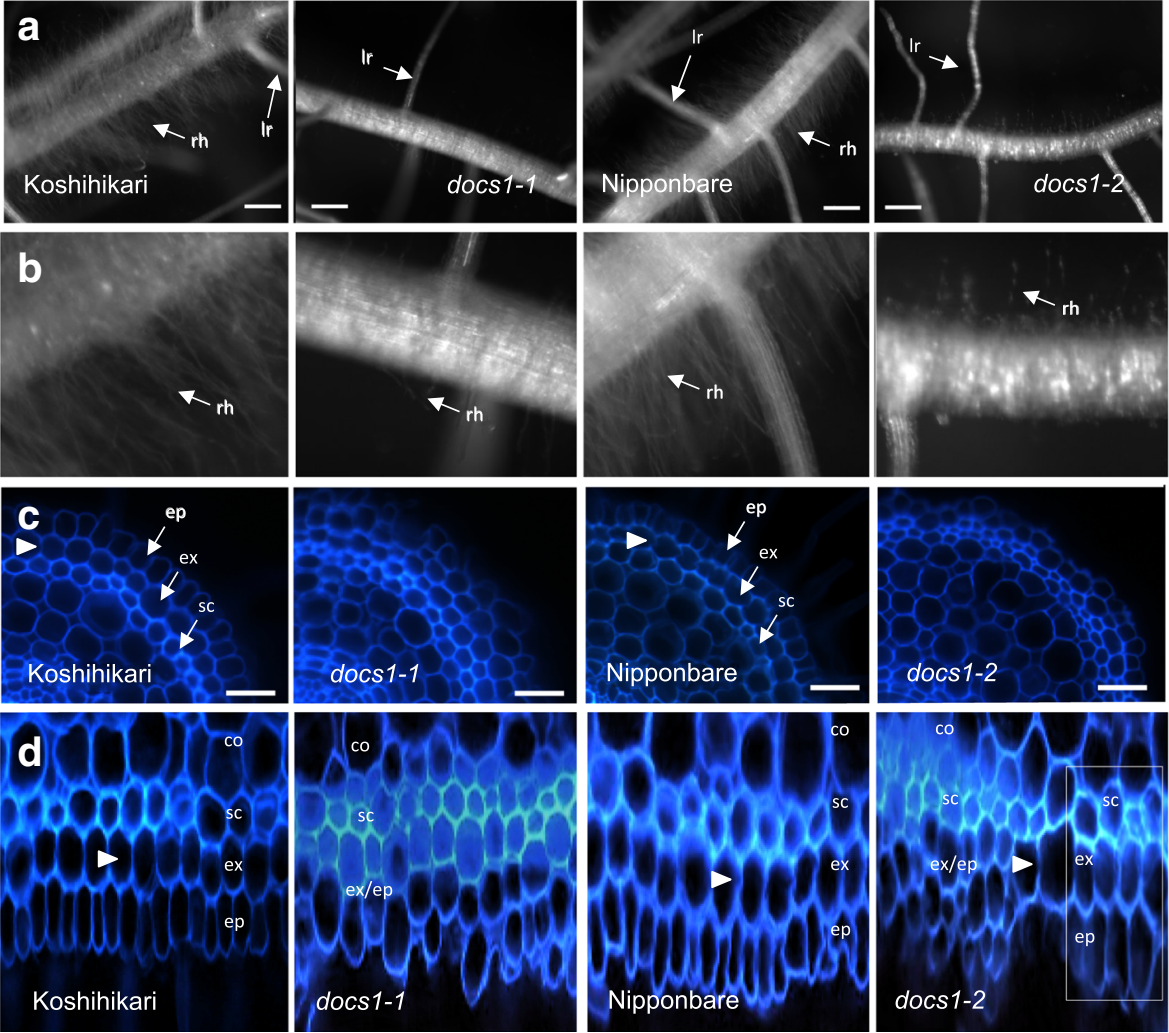
*docs1–1* and *docs1–2* root mutant phenotypes compared to their respective Koshihikari and Nipponbare controls. **a** Root hairs of *docs1–1* and *docs1–2* mutant roots are much shorter and less abundant than their respective controls. lr, lateral root; rh, root hairs. Bars 1 mm. **b** Enlarged views of the root hair zones from (**a**). **c** Organization of outer root cell layers (epidermis (ep), exodermis (ex) and sclerenchyma (sc)) are affected in *docs1–1* and *docs1–2* mutant roots as observed on root cross-sections. The exodermis layer is characterized by a decreased of UV fluorescence on their radial cell walls due to Casparian strip presence (white arrowheads). Bars 20 μm. **d** Disorganization of outer layers is highlighted on polar views of the root cross-sections. Full images of these polar views made with the imageJ software (Lartaud et al. 2015) are displayed in Additional file 1: Figure S1. The white box in the *docs1–2* polar view highlights a zone where the three outer cell layers are less disorganized and where the exodermis and epidermis cell layers seem to have differentiated normally. co, cortex

In previous genetic analyses, the *docs1* mutant allele of c68, that will be renamed *docs1–1* hereafter, has been shown to be recessive to the WT *DOCS1* allele. This mutant allele possesses a 1-bp deletion in the tenth exon of the *DOCS1* gene (LOC_Os02g14120, Os02g0236100), which could result in the production of a truncated receptor missing the end of the kinase domain (Huang et al. 2012). In our study, we wanted first to evaluate if gene activity was completely lost in the *docs1–1* mutant. We therefore produced a new mutated allele of the *DOCS1* gene by the CRISPR technology. We targeted this new mutation in the 5′ end of the *DOCS1* cDNA (Fig. 1). The variant obtained presents a deletion of 1 bp at nucleotide 96 after the start codon. This new mutated allele would be predicted to code for very short peptides of 39 amino acids corresponding to the first 32 amino acid of the DOCS1 protein plus 7 amino acids from out-of-frame transcriptional residues. To evaluate if, like *docs1–2*, the *docs1–1* allele was a loss-of-function, we compared *docs1–1* and *docs1–2* phenotypes. First, the root hair morphology of these mutant plants was observed under a binocular microscope. As seen previously for *docs1–1* plants compared to their Koshihikari WT control (Huang et al. 2009; Huang et al. 2012), the *docs1–2* mutant plants possessed less and shorter root hairs than their Nipponbare WT control (Fig. 2a-b). Second, we compared root anatomical parameters. Again, like in *docs1–1* plants, morphological changes of the outer cell layers were observed in the *docs1–2* lines (Fig. 2c-d and Additional file 1: Figure S1). Indeed on root cross-sections 0.5 cm from the apex, and on polar views of these sections, epidermal, exodermal and sclerenchyma of mutant cells are not gathered in 3 well-defined layers. Even if some exodermal cells were identifiable among these disorganized tissues because of auto-fluorescence extinction at Casparian bands, most of the cells forming the root outer cell layers seemed to have mixed identities. Sometimes, the 3 sclerenchymal, exodermal and epidermal cell layers were identifiable among these disorganized tissues (see the white box in Fig. 2e), suggesting that some epidermis cells may have the ability to differentiate into root hair cell types. However, since these well-organized tissue areas are rare, it could explain why the root hairs on *docs1* mutant roots are so scarce. Since these root hair phenotypes have been shown to affect aluminum sensitivity in *docs1–1* (Huang et al. 2009), we also tested the sensitivity to aluminum of the *docs1–2* lines. We used the protocol previously developed by Huang et al. (2009). After 5 days of growth in hydroponic solution, 30 μM of AlCl_3_ was added to the media. The relative root elongation (RRE) of control and *docs1–2* mutant roots was calculated after a 24 h treatment. In concordance with what has been observed previously with *docs1–1* plants, the *docs1–2* line RRE was decreased of 86.5%, in comparison with a reduction of only 66.7% for the WT Nipponbare control roots. These various phenotypic analyses show that the phenotypes observed for *docs1–1* and *docs1–2* mutant plants are identical, indicating that the original *docs1–1* allele is a loss-of-function mutation.

### *docs1* mutants show altered gravitropic perception and response at adult and seedling stages

**Fig. 3:**
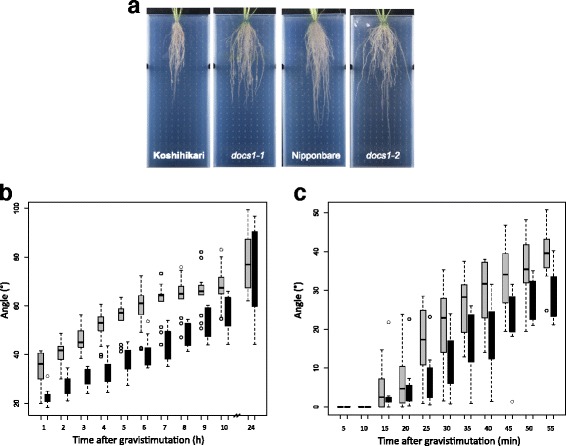
*docs* mutants are affected in gravitropic responses. **a** At 28 days, *docs1* mutant plants grown under hydroponic conditions in rhizoboxes (plexiglas plates filled with 5 mm diameter glass beads, see M&M for details) present a larger root cone angle than their respective controls. **b**, **c** Gravitropic response of *docs1–1* mutant (black) is affected compared to Nipponbare control (gray) seedlings. Seedlings have been grown for 3 days in the dark on MS/2 solid medium before rotating the petri dishes of 90°. Radicle angles have been measured every hour for 10 h, and at 24 h (**b**, *n* = 13 for Koshihikari, *n* = 9 for *docs1–1*), and every 5 min for one hour (**c**, *n* = 8 for Koshihikari, *n* = 7 for *docs1–1*)

We hypothesized that the identity defects and disorganization of root outer cell layers in *docs1* mutant roots could affect root gravitropism. We then grew control and *docs1* mutant plants in rhizoboxes for one month and compared several parameters of their root architectures (see M&M for details). Consistently, larger crown RCA were measured on *docs1* mutant plants than on their respective controls (Fig. 3a). In average, mutant RCA were ~1.5 larger than control ones (~60° vs. ~40°) (Table 1). Since the rhizoscope phenotyping system consists in plexiglass sandwiches filled with glass beads, we wanted to exclude the possibility that the angle phenotype observed could be due to negative thigmotropism response differences between control and mutant plants. We then conducted a second experiment in which rhizoboxes were free of beads (data not shown). Again, mutant plants showed a larger RCA than WT plants, suggesting that gravitropic response more than thigmotropism was affected in *docs1* mutant plants. To measure the gravitropic response, we grew *docs1* and control seedling plants on MS/2 solid medium in petri dishes. After 3 days of growth, petri dishes were rotated of 90° and seminal root tip positions of each seedlings were recorded every hours (Fig. 3b). Results indicate that *docs1* mutants were less sensitive to gravistimulation than control plants. Indeed, *docs1* mutants responded less rapidly than their controls to gravistimulation. Within one hour, the angle formed by control plants was ~35° compared to ~20° for the *docs1* mutants. This marked difference of response was visible up to 10 h after gravistimulation. However, after 24 h, both mutants and controls reached nearly the same angle of curvature. Looking at this response every 5 min for one hour revealed that this response difference was significant as early as 25 min after gravistimulation (Fig. 3c). While most control plants have already turned of ~20° less than 30 min after gravistimulation, *docs1–1* mutant plants reached only ~10° in the same period of time. These data confirm that gravitropic perception and response are affected in *docs1* mutant plants and could explain why GSA is modified in these plants.

**Table 1:** Root cone angle measures in WT and mutant plants grown in rhizoboxes

Plants	Angle (°)	SD	n
Koshihikari	40.1	6.1	>20
*docs1–1*	62.2	7.0	>20
Nipponbare	43.2	6.4	6
*docs1–2*	71.7	10.2	6

### *docs1* mutants present also root cap phenotypes

**Fig. 4:**
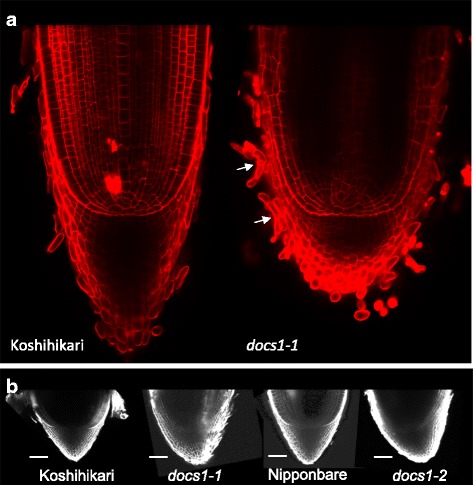
Root cap phenotypes of *docs1* mutants stained with propidium iodide and imaged with a mutiphotonic microscope. **a** Longitudinal view of 3 days old radicle root tips. Tip of the *docs1* root cap seems shorter than control. Arrows point to lateral root cap cells which did not detach from other cells. **b** *docs1* embryo root caps are bent and shifted from the the root central radial axis. Bars 50 μm

Since gravity is primarily perceived by root caps, we then investigated the root cap anatomy and morphology of *docs1* seminal roots (Fig. 4). First, we imaged longitudinal sections of 3 days old radicles under a multiphoton microscope (Fig. 4a). Previous study had already mentioned that lateral root caps were affected in *docs1–1* mutants (Huang et al. 2012). We could confirm that in the *docs1* mutant plants, the lateral root cap cells were more tightly attached to the lateral root caps than in the WT. Moreover, besides this lateral phenotype, *docs1* root cap tips seemed also shorter than WT. To go further into this analysis, we imaged radicle tips of embryos soaked in water for 24 h (Fig. 4b). This developmental stage has been used to avoid any bending artifact of the roots in the course of the experiments. At the embryonic stage observed here, *docs1* lateral root cap cells seem to desquamate on one side of the root cap leading to a stronger fluorescent signal. This phenotype is not observed on WT roots. Besides the lateral root cap detaching cells, *docs1* mutant root cap shapes were also different from WT. Indeed, *docs1* mutant root cap tips were shifted and not aligned on the root central radial axis. Moreover, columella cell files were bent in mutants. These anatomical and structural modifications of *docs1* mutant root cap cells suggest that the *DOCS1* gene could play a role in root cap development during embryogenesis.

## Discussion

Our data show that the loss of activity of the LRR-RLK *DOCS1* gene affects gravitropism perception and response of rice roots at several stages of development. At seedling stage, our data on root cap developmental defect and external tissue disorganization suggest that the early gravitropic response, including gravity perception, could be impaired in *docs1* mutants. At cellular and molecular levels, the timing of events leading to root bending after gravistimulation is well described in Arabidopsis, and rely on the formation of an asymmetrical lateral auxin gradient. This auxin differential flux between the upper and lower side of the root is initiated and maintained by polarized accumulation of several PIN proteins in lateral root cap and epidermis tissues. These differential lateral auxin gradients will activate root cell expansion on the upper side and inhibition of growth on the lower side, resulting in root bending (Band et al. 2012; Rosquete et al. 2013; Sato et al. 2015). In this way, the root cap and epidermal/exodermal morphological disorganizations observed in *docs1* mutant roots could inhibit the early steps of lateral auxin gradient establishment.

In *docs1* mutant roots, molecular activation of the bending response could also be affected. The genomic answer, based on auxin-responsive gene expression, is observed ~15 min after gravistimulation (Band et al. 2012; Sato et al. 2015). This genomic response relies on the regulation of several cell wall-related remodeling genes to trigger cell wall expansion on the upper side of the root to initiate bending. Previous transcriptomic data on *docs1–1* plants have revealed that 1/3 of the 60 genes that were misregulated more than 2-fold in the mutant roots compared to WT were genes involved in cell wall metabolism (Huang et al. 2012). Then, cell wall modifications required for root bending could also be perturbed in the *docs1* mutants.

The gravitropism defects observed in *docs1* mutants lead to modifications of the GSA and result in the development of plant with more opened RCA. The RCA is an important trait in breeding programs, since it determines the soil volume that the plant explores and is potentially correlated to crop production under stress conditions (Uga et al. 2015). Nowadays, the discovery of new genes involved in this trait is then of particular interest to develop new varieties able to cope with changing environmental growth conditions. Radial expansion of the crown roots relies on the suppression of positive gravitropic responses. At cellular level, this response depends, like for gravistimulation response, on auxin redistribution fluxes that will induces differential root side growth. Thus, the same mechanisms responsible for the delayed gravity responses in seminal roots could also affect GSA establishment in crown roots. In *docs1* mutant roots, a decrease in positive orthogravitropic response in crown roots would result in a more radial root system. This would again correspond to a lower polar auxin transport and/or signaling in these roots. However, if these two responses are comparable at the molecular level, the period of time involved in these two processes are different. Indeed, gravistimulated roots will respond within minutes when GSA will take several days to be settled.

To date, and based on our results, our understanding of the exact underlying mechanism of DOCS1 mediated gravity response is still incomplete. Indeed, each of the defects observed in *docs1* mutant plants could participate in the gravity mutant phenotype, namely root cap development, outer root tissue differentiation, and/or misregulated cell wall-related genes. However and surprisingly, despite such morphological and molecular perturbations, *docs1* mutant roots were still able to respond to gravity stimulation, even to a lesser extent. Another important point to take into consideration is that DOCS1 belongs to the SG_II LRR-RLK subfamily and possess 5 LRRs in its extracytoplasmic domain (Dufayard et al. 2017). Other 5-LRR-containing receptors, like the SERKs or SOBIR, have been shown to be co-receptors for other LRR-RLK receptors (Ma et al. 2016). If DOCS1 is also a co-receptor, the different phenotypes observed could be related to its multiple partner functions. This hypothesis will have to be investigated in the future and further genetic and molecular analyses on *docs1* mutant roots will be needed to uncover the function of the *DOCS1* gene in these processes.

## Methods

### Plant materials

The *c68* (*docs1–1*) mutation, which is in a Koshihikari background, has been described previously (Huang et al. 2009; Huang et al. 2012). The new *docs1* allele produced in this study by the CRISPR technology is in the Nipponbare background. The CRISPRsearch algorithm (www.genome.arizona.edu/crispr/CRISPRsearch.html) has been used to define the CRISPR target sequence in the DOCS1 gene (GGCACCTTCGTAGTTGAGCCCCTT). BsaI sites have been added to this sequence for gateway cloning (Invitrogen) into a pUBI-cas9 vector (Miao et al. 2013). Nipponbare calli transformation of this construct has been done through *Agrobacterium tumefaciens*-mediated stable transformation as previously described (Sallaud et al. 2004). Primary plant transformant DNA has been extracted using the standard MATAB protocol (Mieulet et al. 2013). Targeted region of the *DOCS1* gene has been amplified by PCR with the Phusion TaqPolymerase (Thermo Scientific) and the following primers: AAAAAGCAGGCTTGGGGGACCCGCCGCTGTCG and TAACAAAATCCCACGAACGAATCAG. PCR products were sequenced and chromatograms were analyzed manually to identify mutations. The new mutant allele has been named *docs1–2*.

### Phenotyping

#### Root hairs and anatomy defects

After sterilization, seeds were sown on a MS/2 solid medium in sterile petri dishes (Corning, 431.301; 20 cm × 20 cm). After six days of growth in a growth chamber (day/night temperature of 28/25 °C and a 12 h photoperiod, 500μEm-2 s^−1^), root seedlings were pictured under a binocular loop to observe root hair phenotypes. Root tips were also cut and embedded in liquid agarose (3%) for sectioning. After solidification, roots were sectioned (60 μm thin slices) with an Hm650v Vibratome (Thermo Scientific Microm). Root sections were then transferred on glass slides humidified with distilled water. Observations were made with a LEICA DM4500 microscope. Epifluorescence was observed with a specific A cube (excitation in UV, excitation filter: BP 340–380, suppression filter: LP 425). For this experiment, at least two series of 5 seedlings of each genotype have been analyzed (*docs1–1* vs. Koshihikari and *docs1–2* vs. Nipponbare). For embryo root cap observations, after imbibition of dehusked dry seeds in water for 24 h, embryos were excised from the endosperm with a razor blade. For longitudinal observations, seedlings were grown in sterile petri dishes on MS/2 solid medium for 3 days. Before observation, embryos and radicle root tips were put in 10 μM propidium iodide for 1 h with occasional degassing. After rinsing briefly in water, root tip radicles of embryos and seedlings were observed and imaged with a Multi-photon Zeiss LSM 7MP OPO (1100 nm).

#### Aluminum assay

The protocol used has been previously described (Huang et al. 2009). Briefly, plants were germinated and cultivated in 5 l containers on nets floating on a 0.5 mM CaCl_2_ solution (pH 4.5) in a growth chamber (12/12 day length - 21 °C night-28 °C day) for 5 days. Eight containers have been used, each containing 40 seedlings (10 of each genotype analyzed). On day 5, 30 μM of AlCl_3_ hexahydrate solution were added to half the containers. Root length was measured on day 5 before the aluminum addition (excluding plants with very slow germination or stunted growth) and after 24 h of treatment. For both the control and the treated containers, the relative root elongation (RRE) was calculated ((root size after AlCl_3_ - root size before AlCl_3_ treatment) × 100)/ root size before AlCl_3_ treatment). Differences in RRE between the mutants and their respective WT were tested using the contrast method (*P* < 0.05) both for the control and the stressed conditions. The contrasts between the mutants and their respective WT were significant for the stressed conditions but not for the control conditions.

#### Rhizoscope assay

Seedlings were grown under hydroponic conditions in 50 cm × 20 cm × 3 cm plexiglas plates (rhizoboxes) filled or not with 5 mm diameter glass beads in the rhizoscope platform as described in detail in (Bettembourg et al. 2017; Courtois et al. 2013). Each rhizobox contained a grid of nails, which held the root system in place after bead removal. After 28 days of growth, several parameters were measured (number of tillers, length of the longest leaf, maximum root length, depth reached by at least three crown roots and angle of the root cone). A first experiment has been conducted to compare *docs1–1* (>20 plants) to their WT Koshihikari control (>20 plants), and *docs1–2* (6 plants) to their Nipponbare control (6 plants). In a second experiment, rhizoboxes with and without beads were used. In this experiment, six *docs1–1* and six WT Koshihikari control plants were phenotyped for each treatment.

#### Gravitropic assay

Seedlings were grown on a MS/2 solid medium in sterile petri dishes for 3 days in the dark. Then, a 90 degree-rotation was performed on the petri dishes. In two different experiments, the radicle tip positions were marked (i) every 5 min for one hour, or (ii) every hour for 10 h and at 24 h. At the end of the experiments, the plates were scanned. The scanned images were then analyzed with ImageJ to measure the angle of the root tip relative to the original position at t0. Three experiments with ~10 seedlings of each genotype have been performed. Seedlings whose roots did not response in the first hour of the experiments have been discarded from the analysis.

## Additional file

Additional file 1: Figure S1. Polar views of *docs1* root cross sections made with the imageJ software. Only outer root cell layers (epidermis (ep), exodermis (ex) and sclerenchyma (sc)) are affected in *docs1–1* and *docs1–2* mutant roots compared to their respective Koshihikari and Nipponbare controls. No difference is observed in other tissues like endodermis (en) or cortex (co). The white box in the *docs1–2* polar view highlights a zone where the three outer cell layers are not disorganized. (JPEG 367 kb)

## Abbreviations

CRISPR: Clustered Regularly-Interspaced Short Palindromic Repeats
DOCS1: Defective In Outer Cell Layer Specification 1
GSA: Gravitropic Set-point Angle
LRR-RLK: Leucine-Rich Repeat Receptor-Like Kinase
PAM: Protospacer Adjacent Motif
RCA: Root Cone Angle
RGA: Root Growth Angle
RRE: Relative Root Elongation
WT: Wild Type
