# Supplementary figures and images for: Root cone angle is enlarged in docs1 LRR-RLK mutants in rice
Source: Rice (N Y). 2017 Dec 15;10:50. doi: 10.1186/s12284-017-0190-1 (PMC5732118; doi:10.1186/s12284-017-0190-1)

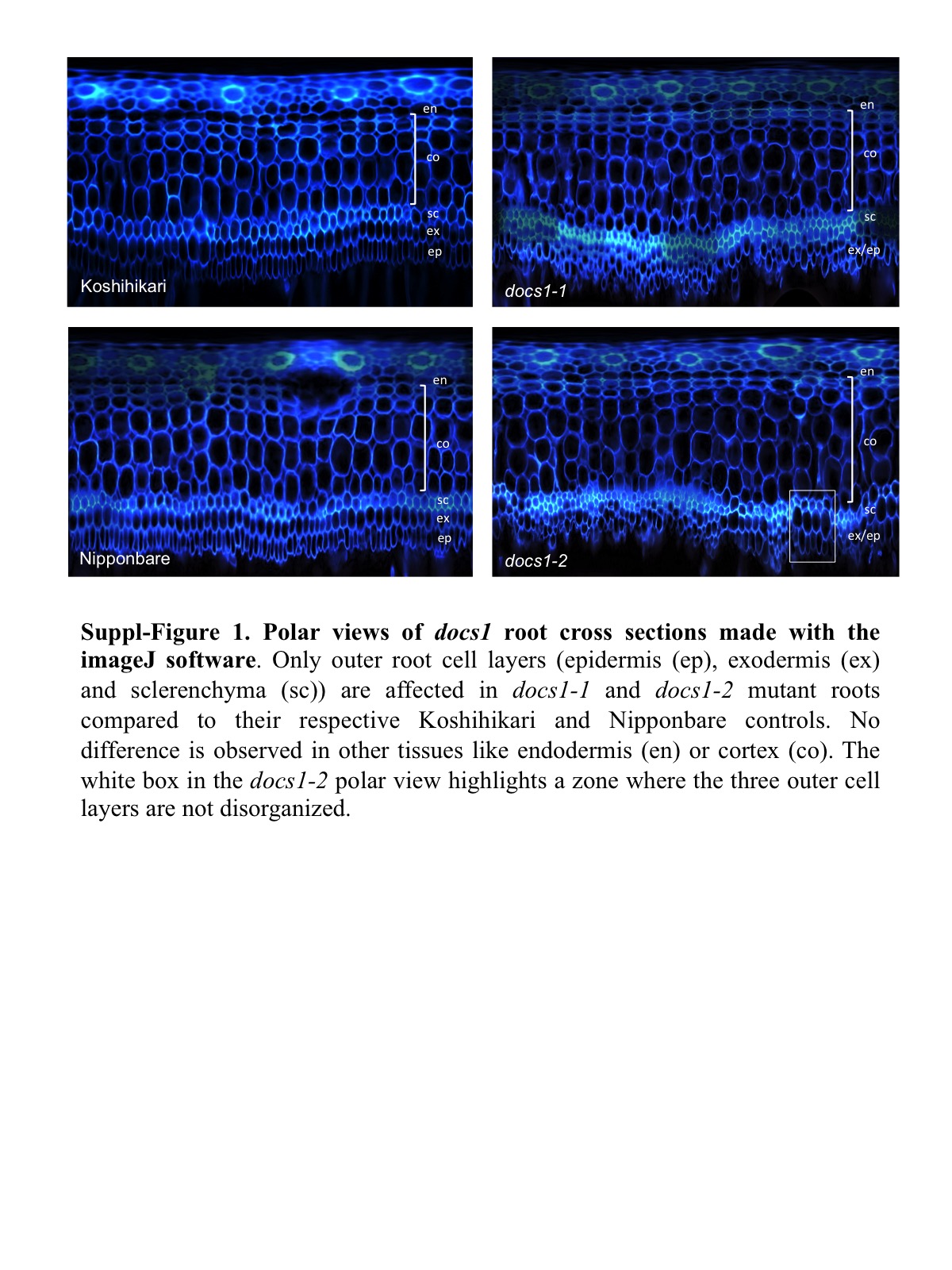

Supplement: Additional file 1: Figure S1. — Polar views of docs1 root cross sections made with the imageJ software. Only outer root cell layers (epidermis (ep), exodermis (ex) and sclerenchyma (sc)) are affected in docs1–1 and docs1–2 mutant roots compared to their respective Koshihikari and Nipponbare controls. No difference is observed in other tissues like endodermis (en) or cortex (co). The white box in the docs1–2 polar view highlights a zone where the three outer cell layers are not disorganized. (JPEG 367 kb) [file 12284_2017_190_MOESM1_ESM.jpg]
